# Forensic Applications of Markers Present on the X Chromosome

**DOI:** 10.3390/genes13091597

**Published:** 2022-09-07

**Authors:** Fernanda M. Garcia, Bárbara G. O. Bessa, Eldamária V. W. dos Santos, Julia D. P. Pereira, Lyvia N. R. Alves, Lucas A. Vianna, Matheus C. Casotti, Raquel S. R. Trabach, Victor S. Stange, Débora D. Meira, Iuri D. Louro

**Affiliations:** 1 Núcleo de Genética Humana e Molecular—Departamento de Ciências Biológicas—Universidade Federal do Espírito Santo, Vitória 29075-910, Espirito Santo, Brazil; 2 Laboratório de DNA Forense—Polícia Civil do Espírito Santo, Vitória 29045-402, Espirito Santo, Brazil

**Keywords:** X chromosome short tandem repeats (X-STRs), X chromosome markers, forensic genetics, population genetics, kinship testing

## Abstract

Microsatellite genetic markers are the gold standard for human genetic identification. Forensic analyses around the world are carried out through protocols using the analysis of STR markers in autosomal chromosomes and in the Y chromosome to solve crimes. However, these analyses do not allow for the resolution of all cases, such as rape situations with suspicion of incest, paternity without a maternal sample for comparison, and biological traces with DNA mixture where the profile sought is female, among other situations. In these complex cases, the study of X-chromosome STR markers significantly increases the probability of identification by complementing the data obtained for autosomal and Y-chromosome markers, due to the unique structure of the X chromosome and its exclusive method of inheritance. However, there are currently no validated Brazilian protocols for this purpose, nor are there any population data necessary for statistical analyses that must be included in the issuance of expert reports. Thus, the aim of this article is to provide a literary review of the applications of X-chromosomal markers in population genetics.

## 1. Introduction

The X chromosome is considered one of the most stable nuclear chromosomes, presenting a size length of approximately 155 million base pairs (Mb), accounting for nearly 5% of the human genome [1,2,3].

The X chromosome has many characteristics that are not shared by its counterpart, the Y chromosome [2]. In males, the heterogametic sex, there is a single copy of the X chromosome and a single Y chromosome [4], while in females, there are two copies of the X chromosome [1,4,5].

These mammalian sex chromosomes are believed to have evolved from an ordinary pair of autosomes, referred to as the ancestral protosex chromosomes. The proto-X and proto-Y underwent a series of deletion/addition events during evolution and became the modern X and Y. Additionally, it is believed that a mutation in a sex-determining locus (SRY) is responsible for triggering an evolutionary process of stepwise loss of recombination between the ancestral autosome pair, creating an X-specific region and a Y-specific region in the proto-Y (MSY) [1,5].

The X-chromosome has an exclusive inheritance pattern, according to gender. In the male gender, the X chromosome is (almost entirely) transmitted to females as an unchanged block, while in females, the two X chromosomes present can recombine during meiosis in the same way as the autosomes, and the new reshuffled chromosome is then transmitted to female and male offspring [2,6,7]. However, some recombination between the X and Y chromosomes in males is necessarily retained, ensuring proper segregation in meiosis. This recombination only occurs within two homologous sub-telomeric zones of the chromosomes, known as the pseudo-autosomal regions (PAR) [5,6]. In humans, there are two of these regions, known as PAR1 and PAR2, and it is believed that the two PARs present very different origins and properties [5].

To equalize gene expression between males and females due to type XX/XY chromosomal heteromorphism, one of the two X chromosomes in females is randomly inactivated during early embryonic development. This inactive X chromosome is called the Barr body. However, in the female germline, the inactive X is reactivated before meiosis, which ensures that all oocytes will inherit an active X chromosome [1]. The initiating event to conversion from active X chromosome to inactive X chromosome is the expression of the long noncoding (lnc.) RNA, the X-inactive specific transcript (XIST). The XIST RNA molecules coat the inactive X chromosome and recruit chromatin modifiers that lead to the silencing of most of the genes along its length [8]. However, the inactive X chromosome is not completely silent because many other genes continue to be expressed from this chromosome, in addition to the active X chromosome [4].

These X-chromosome specific properties make it a powerful complementary tool in forensics and population genetics, helping to solve complex cases, such as missing persons, incest, immigration, deficiency, paternity, and other issues, as well as for use in other research areas, such as human evolutionary studies and medical genetics [2,7,9].

Thus, the main objective of the present work is to review the advances and applications of markers present on the X chromosome related to population and forensic genetics.

## 2. X-Chromosome Markers

Over the years, the particular structure and singular properties of the X chromosome have highlighted the use of X chromosome markers in forensics and population genetics analysis. The knowledge obtained from these markers can be used in isolation, or in a complementary analysis of the information from autosomal and Y chromosome markers, or even from the mtDNA [2,10]. Therefore, these markers are a useful tool for obtaining an accurate interpretation, for example, when there are DNA mixtures. Here, we describe the main X chromosome markers and the specificity of each one.

### 2.1. X-STR

Short Tandem Repeats (STRs) are DNA sequences with repeat units that are 2bp to 7bp in length, which are widespread throughout the human genome [11,12]. STRs show abundant variability among individuals in a population and have become useful for different purpose, including genetic mapping, disease diagnosis, linkage analysis and, in particular, for human identification [12,13].

Gomes et al. (2020) [2] suggest a number of characteristics that makes STRs the preferential markers in human identification analysis. First, they are highly polymorphic, resulting in high discrimination capacity between individuals; second, they are rapidly and easily analyzed using PCR-based technology and capillary electrophoresis automated fluorescent detection; third, STRs exhibit a multiplex generation capability, with short amplicon lengths for degraded DNA.

Furthermore, there is a Short Tandem Repeat DNA Internet Database (http://www.cstl.nist.gov/biotech/strbase/, accessed on 1 July 2022) [14] compiled and maintained by The National Institute of Standards and Technology (NIST) since 1997. This database is an important resource that combines information from the literature with commonly used technologies and materials for STR DNA markers [13].

STR markers are not only autosomal, but also occur on the X and Y chromosome [15]. The utilization of X chromosomal STRs (X-STRs) can be valuable, as it may be used as an additional data source in complex cases where the analysis of autosomal markers is not informative. Therefore, X-STR can efficiently generate more information than autosomal STR, particularly in complicated kinship analysis [15,16,17,18].

X-STRs are highly standardized with numerous markers, methodologies, and databases, and there are commercial kits available on the market, for instance the Investigator Argus X-12 QS Kit (QIAGEN, Hilden, Germany), that permit the analysis of 12 X-STR markers [2,17]. Moreover, Szibor et al. (2006) [19] created an X-STR online database (www.chrx-str.org accessed on 1 July 2022) including 55 X-STR so far, although only 15 X-STR have complete and extensive information available (DXS6789, DXS6809, DXS7132, DXS7133, DXS7423, DXS8378, DXS9902, DXS9898, DXS10074, DXS10101, DXS10134, DXS10135, GATA172D05, GATA31E08 and HPRTB) [18].

### 2.2. X-SNPs

Single nucleotide polymorphisms (SNPs) are a single-base sequence variation highly abundant in the human genome. They may be present not only in genes (exons and introns), but also in the noncoding regions of the genome. SNPs are the most common type of genetic variation and may be used to aid in distinguishing individuals from one another. These polymorphisms are being used for linkage studies to track genetic diseases, for human evolutionary history studies, and they have also been considered as potential genetic markers by the forensic community [20,21].

According to Tomas et al. (2010) [22], the main advantages of including SNP in forensic analysis are the low mutation rates and the fact that it can be typed from small amounts of DNA, making them particularly useful in degraded DNA and difficult samples. However, there are significant disadvantages for SNPs when compared to STR markers. For example, to obtain equivalent match probabilities, it is necessary to analyze 40–60 loci of SNPs compared to 13-15 STR. Moreover, when there are sample mixtures, the interpretation by SNPs typing can be very difficult due to a limited number of alleles compared to multi-allelic STR markers [23]. Therefore, SNPs markers are most likely to have potential future in forensic application only to estimate ethnicity and to predict phenotypic characteristics [21,23].

Some efforts were made to analyze X-chromosomal SNP genotyping (X-SNP) in forensic cases to complement the analysis of autosomal, Y-chromosomal, and mitochondrial markers, especially in deficiency cases [16,22,24]. Although the use of X-SNPs in special relationship testing is promising, the interpretation is very complex and difficult, especially in mixed samples. Moreover, to elevate the combined power of discrimination, an increased number of X-SNPs are required, thus limiting the application of these markers in forensic cases, thus justifying the lack of interest in its use [2,22,24].

The FORensic Capture Enrichment SNP (FORCE SNP) panel, developed by Till-mar et al. (2021) [25], is a complete SNP panel applied in forensic cases. In this panel, clinically relevant markers are excluded, avoiding DNA database privacy concerns. It contains all relevant SNP markers for forensic applications, such as identity, ancestry, phenotype, and X and Y chromosomal SNPs. In addition, it features a new set of kin SNPs for inferring distant relationships (up to 4th degree relationship, with high statistical significance).

The FORCE panel includes features such as a relatively small size and a minimal number of primers/probes per reaction to reduce enrichment costs. The versatility of this panel is confirmed by the possibility of using enrichment methods such as hybridization capture, PEC, and multiplex PCR, allowing for the analysis of degraded samples. The inclusion of X-SNPs in the panel is due to their informative value of kinship for cases of specific X-chromosome inheritance, further enhancing the panel’s analytical performance [25].

### 2.3. X-INDELs

Insertion Deletion Polymorphisms (INDELs) are biallelic markers that combine the interesting aspects of both SNPs and STRs. INDELs have low mutation rates, they are widely spread throughout the genome, including along the X and Y chromosome, they have short amplicon size, making them easy and inexpensive to analyze, and they can be representative of differences between geographically distinct populations [26,27,28,29].

INDELs have received less attention than SNPs in forensic studies, but they may also be an important marker to complement STR analysis, increasing the identification success rating in cases of degraded DNA [30]. Two main studies [31,32] call attention to the applicability of INDELs that may be underutilized for genetic studies in forensic science [28].

Over the last few years, there has been a growing trend toward examining X-chromosome INDELs markers, mainly in the field of evolutionary anthropology, to assess the admixture of population and kinship investigations with deficient relationships [29]. Despite exhibiting greater efficiency than the markers on the autosomal chromosome, the X-INDELs are still limited by their lower discriminating power compared to X-STR [28].

## 3. Population Genetics

Due to its unique inheritance patterns, the use of markers for the X chromosome in population studies has been more explored in recent decades. Features such as a lower recombination rate and a lower mutation rate result in faster genetic drift. Consequently, this makes linkage disequilibrium (LD) and the population structure of the X chromosome stronger. In women, the X chromosome transfers two-thirds of its genetic inheritance. Thus, understanding the genetic diversity of a population can help in demographic studies involving migration and sexual reproduction patterns [9].

X-STR markers are preferentially used in population analyses because they are highly polymorphic, technically easy to analyze, and they exhibit the ability to generate multiplex STRs with smaller amplicons [2]. To use them in these cases, specific knowledge about the frequency of alleles and haplotypes, as well as genetic linkage status and LD, is required. Genetic linkage assesses the co-segregation of loci located nearby in a pedigree, while linkage disequilibrium assesses the co-segregation of alleles at the population level [33]. Since population data are fundamental for forensic investigations, it is of great importance that there is more information compiled and organized on the X-STR markers of populations.

For forensic and human identification studies using molecular markers, linkage disequilibrium (LD) data between the tested loci is used to ensure the reliability of the results. To achieve this, LD studies regarding the recombination of data between loci are carried out, such as those of Phillips et al. (2012) [34], who evaluated the genetic distance of centimorgans (cM) to infer recombination rates at the loci of different STRs of the X chromosome, required for kinship tests due to the density and uneven distribution of the markers.

The main markers used for population and forensic studies are grouped into 4 linkage groups (LG): LG1-DXS10148, DXS10135, and DXS8378; LG 2-DXS7132, DXS10079, and DXS10074; LG 3-DXS10103, HPRTB, and DXS10101; and finally LG 4-DXS10146, DXS10134, and DXS7423. Each set of three markers is defined as a haplotype [34].

To better understand the genetic landscape of a given population, several studies were carried out using X-STR markers, as was the case with Ferragut et al. (2021) [9]. They evaluated the genetic diversity of a Western Mediterranean population using 12 X-chromosome markers included in the Investigator Argus X-12 kit (Qiagen GmbH, Hilden, Germany). Based on the X-STR analysis, it was possible to suggest a gender-biased migration rate, confirming the predominance of patrilocality in this area. In 2019, a similar study was conducted by Garcia et al. [35], with the aim of building a database of X chromosome markers in the Argentine population. The Investigator Argus X-12 kit was also used in this study, and 914 complete haplotypes were obtained for the markers included in the kit. Knowing the uniqueness provided by the X-STR markers, several research groups conducted studies to assess the population distribution of countries such as Brazil, Switzerland, Italy, India, Croatia, and countries on the African continent, among others [9,15,36,37,38,39,40,41,42,43,44,45,46,47,48,49]. Table 1 lists some population studies that explore X-STR markers, published in the last ten years.

In the cases mentioned above, the amplification kit chosen was the Investigator Argus X-12 kit (Qiagen GmbH, Hilden, Germany), being the most widely used amplification system. However, the literature reports that the X-STR loci are found in four linkage groups, whose combined system performance may be the identification of less than 12 independent X-STR loci. In addition, many forensic types of X-STR rely on self-designed, non-commercial multiplex assays. Because of this, there was a need for the development of a multiplex system including more unlinked X-STRs [6].

In this sense, research groups focused on the development of amplification kits with a greater number of markers. Zhang et al. [37] developed and validated the TYPER-X19 multiplex amplification system, consisting of 18 STR loci on the X chromosome (DXS9895, DXS8378, DXS9902, DXS6810, DXS7132, DXS10079, DXS6789, DXS7424, DXS101, DXS67683, DXS7, DXS7 GATA165B12, DXS10103, HPRTB, GATA31E08, DXS8377, and DXS7423) and amelogenin. It was possible to verify that the TYPER-X19 system was sensitive, reliable, and efficient for use in forensic approaches. Another amplification system was developed by Xiao et al. (2021) [38], called The Microreader™ 19X Direct ID System, containing 19 X chromosome markers (DXS6795, DXS9907, DXS6803, GATA172D05, DXS6807, GATA31E08, DXS7423, DXS6810, DXS101, DXS9902, 33S6871, DXS688, DXS10162, DXS6809, DXS10135, HPRTB, GATA165B12, and DXS10079) including amelogenin. The results showed that the system is efficient and reliable.

In-house multiplex kits containing many highly polymorphic markers are also used. The GHEP-ISFG decaplex was developed by Gusmão et al. (2009) [50], and it contains 10 X-STR markers: DXS8378, DXS9902, DXS7132, DXS9898, DXS6809, DXS6789, DXS7133, GATA172D05, GATA31E08, and DXS7423. The study analyzed the allele frequencies of Iberian and Latin American populations, and the kit demonstrated efficiency and robust performance.

Allele frequency differences at each locus vary across different populations. DXS10146 and DXS10135 markers presented 38 alleles, revealing the most polymorphic alleles. The most informative locus among the population studies mentioned is DXS10135, shown in 11 different studies [9,15,35,39,40,42,43,45,46,49]. This is due to the high PIC (polymorphism information content) value of this locus in the different populations [51].

X-STR allele sequence variation data were found primarily at the DXS10134 locus, showing two more repeats [GAAA] in the GRCh38 and at the DXS10146 locus, with four sets of nucleotide differences, including an extra T nucleotide in the GRCh37 assembly in X:149584331 and an extra repeating unit [AAAG] in GRCh38 [51].

Although X-STR markers are extremely efficient for population analyses, the absence of a database for the collection, storage, and use of frequencies of X STR alleles or haplotypes is a factor that renders their use difficult [2,6]. To date, the main ways to obtain this data are through consultation of publications in the PubMed database, conference proceedings of the International Society for Forensic Genetics (ISFG), and the Forensic ChrX Research website. In some countries, an organized database is available for consultation regarding haplotypes, as is the case of the Brazilian Genetic Bank of the X Chromosome (BGBX) [6,44]. 

Moreover, several countries do not have data compiled and stored for X-STR. The greatest scarcity is seen in areas such as Sub-Saharan Africa and the Americas (except for the USA, Brazil, and Argentina). On the other hand, China has made significant progress in this regard in recent years. The country holds a large amount of information and studies about the X-STR marker for its population [2].

The use of X-STR markers in population studies is promising, but to advance the issue, further studies on the frequency distributions of haplotypes, mutation rates, and LD are essential. Considering these observations, it will be possible to build an effective human X-STR database that contains comprehensive data from different populations in different parts of the world.

## 4. Forensic Applications

### 4.1. Parenthood Testing

The inference of genetic kinship between two individuals has been a subject of great theoretical and practical interest in the forensic field [52]. With current technological advances, a specific demand for kinship testing is expected to arise where only remote relatives are available for testing [16], and there are a multitude of applications for paternity testing, such as the clarification of bilateral relationships [53], determination of kinship in immigration proceedings, and identification of parental lines [54].

In this way, the paternity test (PT) becomes a very important instrument for the advancement of forensic genetics in a wide spectrum of activities. However, it is worth noting the comparison of the main differences between the PT and the maternity test (MT), because, as for the PT, there is an understanding that the involvement of the mother’s genotype generates an increase in the power of identification of the biological father [53]. However, in the absence of maternal data, the exam may be inconclusive [55].

Insertion of markers based on STR sequences and mitochondrial DNA sequence variations linked to the analysis of sex chromosomes (X and Y) provide greater PT efficiency, with respect to autosomal markers [16,55]. Owing to the inheritance pattern of Chr-X, in which the daughter receives the unaltered paternal X chromosome, Chr-X markers have a high power of exclusion [17]. The X-STRs exclusion power is due to the difference in the number of alleles when compared to autosomal alleles in male individuals [44].

Because of these unique characteristics, X-STRs can satisfactorily complement cases in which the analyses of autosomal STRs are not sufficiently informative, as in father-daughter duo cases. Therefore, in these cases, the analysis of X chromosomal markers can be more informative than autosomal markers.

X-STRs can also be highly informative in cases of father-daughter paternity, where the alleged parents are father and son, as the analysis of autosomal STRs would be inconclusive due to the sharing of alleles. X-STRs inherited from their respective mothers and not shared with each other are very useful in such cases.

In addition, X-STRs can be used in cases of sisters or half-sisters whose common relative is the father. It is possible to observe a greater resolving power, since both, being daughters of the same father, necessarily share the same alleles.

Autosomal DNA markers can pose difficulties when they are physically close to each other on the same chromosome. For these reasons, it is worth highlighting the importance of software, such as FamLinkX, that implements a new algorithm for probability calculations that account for linkage, linkage disequilibrium, and mutations [56,57].

For this reason, such software becomes highly sought after among forensic users as more and more ChrX markers become available [57]. This is justified by its usefulness in calculating case-specific likelihood ratios for two (or more) hypotheses with observed DNA data for a pair of linked DNA markers. In also performs simulations for two or more pedigrees (hypotheses) and analyzes cases that give rise to complex pedigrees. In summary, such compilations of functionalities are now widely available, and are free of charge [34]. 

Moreover, following practices established through adoption and further characterization of X-STR typing and application will promote the development of additional tools, such as software that provides functions for the likelihood calculation of family relationships/pedigrees using X-chromosomal genetic marker data to facilitate their implementation into additional laboratories, providing a rich area for the future of forensic research.

Finally, autosomal STR typing is likely to remain the gold standard for the forensic laboratories well into the future, and X-STR markers have proven to be useful complementary tools in the forensic armory.

### 4.2. Incest

Incest is usually defined as mating between first-degree relatives, (such as father-daughter, mother-son, or brother-sister), who have 30–50% of their genes in common [58,59]. This definition, however, may be expanded with the addition of sexual activity between uncle-niece, grandfather-grandchild [60,61].

Children of consanguineous parents can inherit two alleles identical by descent (ibd) at any locus, show an increase in homozygous genotypes, and are at greater risk for autosomal recessive diseases [62]. Decreased population heterozygosity over the generations is expected in cultures which encourage consanguineous marriages between specific blood relatives (e.g., uncle-niece) [63].

In Brazil, as in many other countries around the world, incest in itself is not a crime. However, in cases where violence or serious threats are used, in which the act is performed with children under 14 years of age, with someone who, due to illness or mental disability, does not have the necessary discernment to perform the act, or who, for any other reason, cannot offer resistance, sexual activity can be considered the crime of rape, or rape of a vulnerable person. In these cases, legal interest arises, given the criminal nature of the act, and there is a need to make use of forensic DNA tools.

Vaginal and oral swabs are commonly collected shortly after the events when incestuous criminal activity is suspected, which may allow for the recovery of spermatic material from the suspect and the comparison of genetic profiles (victim-aggressor).

Child sexual abuse is a global public health concern considered by World Health Organization (WHO) as a silent heath emergency [64]. Victims of incest usually do not talk about the situation due to embarrassment, guilt, and fear. Thus, incest cases are rarely reported [65]. Moreover, the efforts of families to cover up incest cases is a well-known reality [63]. Thus, in many rape cases, sperm is not available from vaginal swabs, and the only resulting genetic evidence may be the products of conception [59]. Therefore, in cases where rape leads to pregnancy, it is possible to compare the genetic profiles of the alleged father, mother, and fetus.

In many circumstances, DNA profiling of autosomal STR loci can be reliably used for solving criminal and paternity cases focused on males [66]. Nonetheless, in those cases involving close blood-relatives as putative fathers, the exclusion power of autosomal STRs is considerably reduced, and ChrX (Chromosome X) STRs may be most appropriate [16]. For example, if two alleged fathers are father and son, ChrX markers would be more efficient than autosomal STRs, since father and son do not share any X-chromosomal alleles idb [63]. In some criminal paternity investigations, the high rate of homozygosity displayed by the child may raise the suspicion of an incestuous situation [2].

The analysis of the ChrX STR profile, in the case of a daughter, is quite informative, even in the absence of the genetic profile of the father. When the father of the daughter is also the father of the mother (father-daughter incest), the child will either be homozygous for all ChrX STR markers or will present the same genotype as the mother [2].

Considering that ChrX is maternally transmitted, the analysis of its STR is not useful in the case of criminal paternity tests in which the child is a boy, since he inherited a Y chromosome from his biological father. However, it can serve as a supplement in criminal maternity test cases.

The estimated frequency of incest ranges from 0.5 to 2%, [67,68,69] but estimates vary by definition and the method of determining cases [70]. Most victims of incest are minors [65], which ultimately makes the social and psychiatric consequences more serious. Thus, it is imperative that public authorities raise awareness in these cases and adopt multidisciplinary and specialized protocols for monitoring victims, especially younger ones, aiming at treatment and full rehabilitation [65].

### 4.3. Complex Cases Using X-STRs

In the last decades, autosomal STR markers have become the best option for most cases of genetic identification, paternity testing, and other kinship analysis. Despite their reliability and high power of discrimination, in some particular cases, autosomal markers provide little information, even with a high number of polymorphisms typed [71]. In these cases, the use of Y-STRs and X-STRs as additional markers by recombination [17,72], could provide more strength to the genetic evidence due to its inheritance characteristics and different recombination patterns [2]. Additional genetic information can increase the statistical values of true parental relationships in analysis and reduce the chances of false attributions [73].

ChrX markers have been rarely employed in forensic practices, although gonosomal markers are especially efficient for solving deficiency cases [74]. X-STRs are particularly useful in complex kinship cases, where just a few and/or distantly related individuals are available for genetic analysis [71], especially when the mother is absent. They are also used in some missing persons/mass disaster situations to identify victims, when direct reference samples are not available and biological relatives must be used [75]. Complex analysis involving singular materials, such as DNA from exhumed bones or historical samples (small number of low size STRs) [74] could also be aided by X-STRs data.

The scenarios when the presumed father is not available for genetic analysis using X-STRs is the most common type of complex kinship testing regarding financial inheritance disputes to prove the affiliation to the deceased alleged father [71]. The father will convey his ChrX copy to daughters only, and all sisters will share at least one allele per locus for the ChrX. In this context, the investigation of sisters or stepsisters can exclude paternity, even if the DNA of the parents is not available, by genotyping the putative grandmother [16].

X-STRs may also be a better choice in cases where the genetic material from different individuals is mixed. The male hemizygous status for X-STRs makes these markers more advantageous compared to autosomal markers [74]. In cases of abortion involving a female fetus, the DNA of the embryo and the mother are mixed, in which case, it is possible to perform a paternity test on the fetus, as alleles not shared with the mother can be analyzed [74].

Thus, X-STRs markers can be useful for any parent–child relationship that involves at least one female [76]. However, for closely linked markers, it is advisable to consider linkage and LD for the most precise likelihood calculation [17].

In that regard, Bini et al. (2019) [76] evaluated possible mutational and recombination events for X-STR markers using Investigator Argus X12 kit in Italian pedigrees. In order to explore the segregation stability, three-generation families (grandpa-mother-son) and two-generation families (mother-sons, father-daughters), for a total of 269 pedigrees, were analyzed, and calculations to estimate the recombination fractions between pairs of markers and mutation rates were performed [76].

It is important to underline the significance of larger databases to enhance the estimation of haplotype frequencies, more software packages for kinship evaluations of ChrX transmission [2], and new, tested, and optimized X-STRs markers for kinship analysis. To support further development of X-STRs, the studies conducted presented the discovery of novel X-STRs markers and multiplex systems which are highly promising for forensic use [49,66,77,78] and the extension of local haplotype data through studies of the viability and discriminatory power of X-STRs [9,40,79,80,81]. Despite more than 20 years of usage and X-STR research in forensic genetics, there is still a continuous demand for high-quality genetic data to support new studies and expand the application of these gonosomal markers.

## 5. Future Directions and Conclusions

The need to resolve sexual assault crimes and to analyze kinship scenarios in areas such as immigration, paternity, missing persons, and mass disasters will remain an important part of the impact forensic science has on society well into the future, especially as the questions surrounding such situations become more complicated and new technologies, such as next generation sequencing, make laboratory implementation of X-STR marker systems more accessible [6,58].

Regardless of the increasing use of SNPs and INDELs markers in forensic cases, mainly due to the advancement of Next-Generation Sequencing (NGS) technology, standardization must be advanced, due to the large number of markers necessary to acquire a high degree of discrimination between individuals in a population [2,82]. The special features of STRs have made them the most popular multi-allelic markers adopted as reference loci for the Combined DNA Index System (CODIS), facilitating the worldwide implementation of the National DNA Databases (NDNADs) [20]. Therefore, at least for the time being, STRs will remain the essential and preferable markers used in forensic studies.

The use of X-STR markers is promising, but more studies regarding haplotype frequency distributions, mutation indications, and LD are necessary to insure that the new markers are incorporated correctly into the routine of forensic companies and laboratories that work with DNA identification.

Furthermore, in order to implement X markers, it is necessary to maintain a single database covering different populations.

In order for X-chromosome markers to be used, distribution and frequency studies in different populations must be carried out.

These methodologies may contribute to solving pending complex cases that fit the criteria for the use of X-chromosome markers, increasing the efficiency, quality, and reliability of services offered for identification, paternity testing, and forensics.

## Figures and Tables

**Table 1:** Population studies using X-STR markers, published in the last 10 years.

Population	Kit	Nr Markers	Year	Ref.
**Switzerland**	Investigator Argus X-12 kit (Qiagen)	12	2021	[36]
**China**	TYPER-X19 multiplex assay	18	2021	[37]
**China**	The Microreader™ 19X Direct ID System	19	2021	[38]
**China**	19 X-STR typing system (in-house)	19	2021	[15]
**Western Mediterranean**	Investigator Argus X-12 kit (Qiagen)	12	2021	[9]
**India**	Investigator Argus X-12 QS kit (Qiagen)	12	2020	[39]
**Eritrea**	Investigator Argus X-12 kit (Qiagen)	12	2020	[40]
**Argentina**	Investigator Argus X-12 kit (Qiagen)	12	2019	[35]
**Mexico**	GHEP-ISFG decaplex (in-house)	10	2018	[41]
**Italy**	Investigator Argus X-12 kit (Qiagen)	12	2018	[42]
**Croatia**	Investigator Argus X-12 kit (Qiagen)	12	2018	[43]
**Brazil**	GHEP-ISFG decaplex (in-house)	10	2017	[44]
**Guinea-Bissau**	Investigator Argus X-12 kit (Qiagen)	12	2017	[45]
**United Arab Emirates**	Investigator Argus X-12 kit (Qiagen)	12	2017	[46]
**USA**	15 X-chromosomal STR markers (in-house)	15	2014	[47]
**Japan**	18 X-chromosomal STR (in-house)	18	2013	[48]
**Europe and Asia**	Investigator Argus X-12 kit (Qiagen)	12	2012	[49]

## Data Availability

Not applicable.
